# An Efficient Paradigm for Genetic Epidemiology Cohort Creation

**DOI:** 10.1371/journal.pone.0014045

**Published:** 2010-11-18

**Authors:** Martin Ladouceur, William D. Leslie, Zari Dastani, David Goltzman, J. Brent Richards

**Affiliations:** 1 Department of Human Genetics, McGill University and Jewish General Hospital, Montreal, Canada; 2 Departments of Medicine and Radiology, University of Manitoba, Winnipeg, Canada; 3 Departments of Epidemiology, Biostatistics and Occupational Health, McGill University, Montreal, Canada; 4 Department of Medicine, McGill University and McGill University Health Centre, Montreal, Canada; 5 Twin Research and Genetic Epidemiology, King's College London, London, United Kingdom; University of Michigan, Canada

## Abstract

Development of novel methodologies to efficiently create large genetic epidemiology cohorts is needed. Here we describe a rapid, precise and cost-efficient method for collection of DNA from cases previously experiencing an osteoporotic fracture by identifying cases using and administrative health-care databases. Over the course of 14 months we collected DNA from 1,130 women experiencing an osteoporotic fracture, at a cost of $54 per sample. This cohort is among the larger DNA osteoporotic fracture collections in the world. The novel method described addresses a major unmet health care research need and is widely applicable to any disease that can be identified accurately through administrative data.

## Introduction

Advances in genotyping technology have recently afforded insights into the genetic determinants of common diseases through the use of genome-wide association (GWA) studies [1]. GWA studies have described common genetic variants which are robustly associated with disease, but impart a relatively small risk. Since these risks are small, but appear to be biologically relevant, very large cohorts of disease cases and their controls are required to identify the common genetic determinants that underpin susceptibility to disease.

Although some prospective disease cohorts have collected DNA, there still exists a need to enlarge these cohorts and associated consortia to achieve adequate statistical power to identify the genetic determinants of disease. Population-based longitudinal prospective studies are able to address a myriad of important scientific questions pertaining to the disease under study. However, since modern genetic studies require very large populations of cases, these longitudinal studies may not be able to provide a sufficient number of cases. Now that genotyping technologies and methodologies are in place, novel methods that can efficiently assemble large genetic epidemiology cohorts without having to re-create longitudinal prospective cohorts are required.

In this paper, we demonstrate a novel method to efficiently create a large genetic epidemiology cohort of osteoporotic fractures by combining cases identified in a large administrative dataset using a highly specific case definition with controls from a prospectively followed population-based longitudinal cohort. To our knowledge, this genetic cohort is among the largest osteoporotic fracture cohorts worldwide. While we developed this cohort for osteoporotic fractures, these methods are directly applicable to the assembly of other genetic epidemiology disease cohorts.

## Methods

We identified potential cases for the cohort using the administrative data repository of Manitoba Health. We selected women who had either a surgically repaired hip fracture, or a forearm fracture with orthopedic reduction and/or casting using previously validated definitions [2] between the ages of 45 and 70 years. Women with known risk factors for osteoporotic fractures, indicators of significant comorbidity, or those whose fracture resulted from severe trauma (as indicated by trauma codes) were excluded.

Prior diagnoses were obtained from hospital discharge abstracts and physician claims. We sought information on systemic corticosteroid use from the province-wide pharmacy database which captures virtually all prescription dispensations within the province. This system maintains high-quality data on all dispensations issued to Manitobans, such as drug, dosage, and prescription date, with a high concordance between the prescription claims database and pill counts [3].

Manitoba Health identified all women within 50 km of the city of Winnipeg meeting our case definitions. A letter was sent by Manitoba Health on behalf of the researchers to all subjects inviting them to participate in the study. Individuals not responding to the initial letter of invitation were sent a reminder several weeks later. The researchers were unaware of the subjects' identities until they contacted the research study office. Those subjects agreeing to participate were mailed a salivary collection kit in the regular post after the informed consent process. We sought permission to re-contact individuals who had agreed to participate in the study, and all but a dozen participants agreed. This follow-up could be by phone, post or clinical visit. We do not have permission to re-contact those who did not respond to our initial invitation.

**Figure 1:**
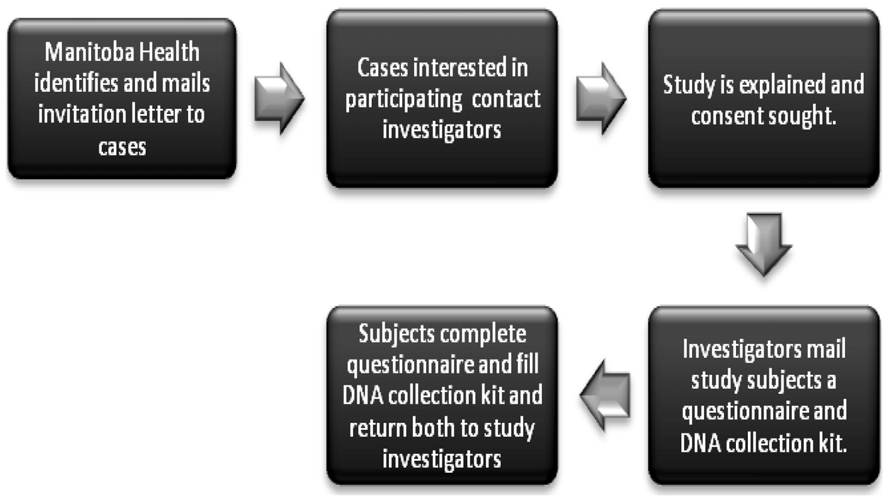
Study Flow for the Manitoba Fracture Cohort.

Individuals were asked to deposit 2 ml of saliva into a collection tube using a standardized protocol (DNAGenotek Oragene, Ottawa, Canada), which was then returned to the study laboratory (**Figure 1**). Saliva can be collected from adults by following the user instructions provided with the Oragene DNA self-collection kit. It is also possible, however unlikely, that some subjects may not be able to produce 2 ml of saliva. In this case, participant were can be asked to perform a buccal swab, which is easier to perform and also available from DNAGenotek. DNA was extracted using the Qiagen QIAmp DNA mini kit as per the manufacturer's instructions. All samples were frozen at -80 Celsius to optimize DNA storage [4]. DNA was quantified using ultra-violet quantification. Salivary DNA collection enjoys several advantages, including ease of collection, shipping and storage, high DNA quality and relatively high DNA yields. See Rylander-Rudqvist et al. 2006 [5], Rogers et al. 2007 [6], and Nishita et al. 2009 [7] for details about quantity and quality of saliva DNA from Oragene kit.

Data collection for the cases and controls included important social and demographic information, and examined general health, lifestyle, and medication use. Data on education level and comorbid diagnoses (self-reported and physician-made) were also collected.

Controls were selected from the Canadian Multicentre Osteoporosis Cohort (CaMos), a longitudinal population-based study following 9,423 randomly selected men and women from the general Canadian population [8]. Controls were women 50 years and older with current contact information, and not reporting a prevalent fracture at baseline or during the 10 years of follow-up. The rates of fracture in women in Manitoba are similar to those in the rest of Canada [9].

Ethics approval: For both CaMos and the Manitoba Fracture Cohort construction, the study was approved by regional institutional ethics review boards, and participants provided written informed consent. The study was reviewed and approved by the Health Information Privacy Committee of Manitoba.

## Results

In total, 7,826 women were identified by Manitoba Health as meeting the fracture case definition and were sent a letter inviting them to participate in the study. Among these potential participants, 173 (2.2%) letters were returned, mostly because the address was wrong, and 11 (0.1%) women were deceased. Of the 7,642 contacted women, 7,149 (93.4%) had sustained a wrist fracture and 502 (6.6%) a hip fracture. Of all the women responding, 99.0% verified that they did indeed sustain the osteoporotic fracture identified in the administrative data. The specificity for the administrative case definition among women contacted for reported hip and wrist fractures was 95.6% and 99.4%, respectively. To date, we have received DNA samples from 1,130 subjects (representing a response rate of 15.8%) of which there were 84 (7.4%) hip fractures and 1,046 (92.6%) wrist fractures. All samples were received within 14 months of initiating the study. The mean age of the women participating from Manitoba was 69.4 years (standard deviation [SD], 8.1) and the vast majority were white. **Table 1** describes the characteristics of the cases and controls. The total cost of establishing the Manitoba-McGill Fracture Genetics Cohort (not including costs associated with DNA extraction) was $54 USD per sample.

**Table 1:** Characteristics of the Manitoba Fracture Cohort Cases and Canadian Multicentre Osteoporosis Study (CaMos) Controls.

Variables	Manitoba Fracture Cohort (Cases)	CaMos (Controls)
Eligible women	7,149	5,566
DNA samples collected	1,130 (15.8%)	2,134 (38.3%)
Age	69.4 [8.14]	71.2 [9.8]
FractureHipWrist	84 (7.4%)1,046 (93.6%)	——
RaceWhiteNon white	700 (96.6%)22 (3.4%)	5,361 (96.3%)205 (3.7%)
Medication use after fractureBisphosphonatesHormone replacement therapyRaloxifeneOthers	184 (60.7%)94 (31.0%)20 (6.6%)5 (1.7%)	————
DNA quantity (ug)	242.1 [215.6]	177.6 [117.4]

Mean [SD] or Frequency (%).

For ethical reasons, the authors were not permitted to contact women who decided not to participate in the study. Consequently, we do not have information concerning the profile (administrative and medical) of the non-respondents, besides their age.

## Discussion

In summary, we have developed a novel paradigm for genetic epidemiologic cohort creation. The method described is cost and time efficient and identified cases with high precision.

Genetic epidemiology cohort creation using the above methods is subject to several limitations. The first is that accurate and valid administrative database codes need to be used to robustly identify cases. Second, the response rate that we experienced in our cohort creation was low. Nonetheless, the sampling frame was exceptionally large and therefore the absolute number of cases collect was high. Indeed, the assembled cohort is now among the largest DNA collections for fracture in the world. Third, the retrospective design of sample collection is susceptible to survivorship bias; wherein only those surviving the fracture would be eligible for enrollment, possibly introducing selection bias. However, 93% of the samples were collected from individuals experiencing a forearm fracture, which has not been associated with increased risk of mortality [10]. Hip fracture, on the other hand, has been associated with 20% mortality at one year [11], however, targeted age range of fracture (45–70 years) in our cohort is relatively low and increasing age is strongly associated with post-fracture mortality; therefore the survivorship bias in our cohort would be reduced. Lastly, it is difficult to estimate the characteristics of the non-responders since we were not allowed to contact them. Fortunately, confounding due to compliance to participation would be unlikely to influence the effect of genetic factors on risk of fracture, since these factors would likely lie in the causal pathway.

In summary, this study design is widely applicable to common diseases easily identifiable through administrative databases, providing a novel paradigm for genetic epidemiology cohort creation. Thus, this method addresses a compelling and unmet health care research need by enabling researchers to efficiently achieve an adequate number of cases for modern genetic epidemiology studies.
